# Patient-to-Patient Interactions During the Pain Management Programme: The Role of Humor and Venting in Building a Socially Supportive Community

**DOI:** 10.3389/fpain.2022.875720

**Published:** 2022-04-27

**Authors:** Katherine A. Finlay, Adam Madhani, Krithika Anil, Sue M. Peacock

**Affiliations:** ^1^School of Psychology and Clinical Language Sciences, University of Reading, Reading, United Kingdom; ^2^Peninsula Allied Health Centre, School of Health Professions, University of Plymouth, Plymouth, United Kingdom; ^3^Pain in the Mind, Independent Practice, The Saxon Clinic, Milton Keynes, United Kingdom

**Keywords:** chronic pain, peer, social support, interpersonal, interaction, conversation analysis, qualitative

## Abstract

**Objectives:**

Social support is most positively perceived when there is an optimal match between a patient's need for communication and the purpose of their interaction. Maladaptive communication patterns may inhibit social bonding or mutual support, negatively impacting clinical outcomes. This study aimed to identify how people with chronic pain naturalistically converse together about their pain in the context of a Pain Management Programme (PMP).

**Methods:**

Seven participants (4 females; 3 males) with ongoing chronic pain who were attending a PMP in a regional hospital in the United Kingdom were audio/video recorded during breaks in their PMP. Interactions were transcribed using Jeffersonian Transcription and analyzed using Conversation Analysis.

**Results:**

Two conversational mechanisms were identified: (1) *Conversational humor*; and (2) *A venting cycle*. Participants used their pain-related experiences construct a motive for a joke, then proceeded to deliver the joke, which initiated a joke return from observers. The sequence was completed by a collaborative punchline. In the venting cycle, an initial complaint was escalated by the sharing of comparable experiences, after which the vent was concluded through a joke punchline, acting as a pivot to move the conversation forwards, terminating the venting.

**Conclusions:**

Humorous interpersonal interactions about chronic pain provided a forum for social support-building within the PMP. Humor was affiliative and built social collaboration, helping individuals to together make sense of their pain in a prosocial atmosphere, approaching pain-related experiences with levity. Patient-to-patient interactions within the PMP were strongly prosocial and inclusive, potentially facilitating enhanced PMP clinical outcomes through collaboration.

## Introduction

The prevalence of chronic pain (CP), persisting for more than 3 months, is rising globally (1). Recent pooled estimates suggest that 34% of adults in the United Kingdom currently experience chronic pain, with chronic widespread pain affecting 5.5 million (12%) of the British population (2). The impact of CP is multifactorial, and aside from its physical and psychological impact, it places significant social burden on People Living with Chronic Pain (PLwCP) and those around them. Factors such as pain-related loss of employment (3) and withdrawal from social engagement (4) are often linked with a significant deterioration in quality of life for PLwCP and their families (5). As a result, the social environment surrounding PLwCP has been widely investigated: where lack of social support is strongly associated with the genesis, maintenance, and worsening of pain symptoms for PLwCP (6), by contrast, positive social relationships and support from family and friends builds work ability, psychological well-being and quality of life (7, 8). The salience of social support when living with CP is therefore clearly evident and it is important that PLwCP are supported to maintain and/or build positive social relationships and social support structures.

Yet recent research has suggested that not all (positive) social support is equal. In line with the optimal matching hypothesis (9), when social support is optimally matched to a person's needs, then their ability to cope with pain is increased (7, 8). Indeed, CP patients report increased satisfaction and lower perceived stress when expressing their feelings to PLwCP who are experiencing similar health challenges (7, 10). However, when there is a mismatch between an individual's health status and the support which is received, this support can be experienced as “toxic positivity” or unsolicited advice (7). This may mean that individuals feel obligated to generate false positivity, expressed and experienced by people with and without CP as inauthentic, reducing individuals' desires to offer or accept social support in the future.

Though social support “matching” undoubtedly confers some benefits, it is possible that the associated outcomes may not be universally positive. Research has demonstrated that when individuals experiencing similar situations express their perspectives collectively, negative emotional contagion may occur (11). This phenomenon reflects the transfer of negative moods and emotions within a group (12), and the automatic synchronization and convergence of such emotions (13). By extension, in the context of chronic pain, this negative exchange spiral of emotions may increase perceived pain symptoms and lead to an overall decline in well-being. Though emotional contagion specifically within chronic pain settings has not yet been investigated, there is undoubtedly a need to better understand the formulation and presentation of person-to-person interactions in PLwCP. Chronic pain management interventions are typically undertaken in group contexts (14) and it is possible that group interactions within these settings may be jeopardizing rather than enhancing outcomes of pain management programmes.

An emerging method to understand emotion and pain experiences is a discursive approach (15). Discursive approaches move beyond pain self-report measures, to look at how “actions in talk” can better depict pain experiences (16). The analytic method of conversation analysis (CA) is a strong discursive approach which gathers information about normative patterns of speech in conversation and it analyses the extent to which deviant cases occur (17). CA is a novel way of understanding medical and clinical interactions (18), and has been widely used in clinical settings to understand communication between patients and clinicians (19–21).

To date, no studies have used CA to assess patient-to-patient interactions; instead, CA research typically focuses on clinician-patient interactions, addressing issues such as resistance (22) and advice giving (23) CA is often exploited to explore conversations on sensitive topics such as weight management (24) and cancer (25), but has not yet been extended to use in chronic pain. Consequently, the application of CA in a chronic pain setting may prove effective in understanding the conversation sequences and discursive organization presenting in interactions between PLwCP. CA may facilitate exploration of the conversational interactions involved in seeking social support from individuals also living with CP within a pain management group setting. This study therefore aimed to apply CA to patient-to-patient interactions in chronic pain management programmes, to investigate the conversational mechanisms involved in seeking mutual support between PLwCP.

## Method

### Design

A qualitative observational study design was employed, using Conversational Analysis in the context of a 6 week Pain Management Programme.

### Participants and Recruitment

This study was approved by the University of Buckingham School of Psychology and Well being Ethics Committee and was granted full NHS ethical approval (REC reference 13/WM/0214). Six weeks before the PMP began, all attendees at three prospective Pain Management Programmes were invited to take part in this study by letter. Inclusion criteria were participants above the age of 18 years, who had experienced chronic pain for at least 3 months, and had attended two or more seminars at the Pain Management Unit, to gain eligibility for PMP attendance. Chronic pain presentation for each patient was confirmed prior to recruitment for this study by objective clinical and subjective pain assessment measures, undertaken during routine clinical care by the specialist Multidisciplinary Team in the Pain Management Unit (comprising Doctors, Pain Consultants, Physiotherapists, Occupational Therapists, Nurses and Psychologists) before referral to the Pain Management Programme.

Preliminary consent to participation was obtained from 19 patients (8 males and 11 females) across three PMPs. Although consent was obtained from 19 volunteers, in only one out of the three PMP groups was consent to filming/audio recording given by all PMP group members. Therefore, to ensure anonymity of non-consenting PMP members, data was only recorded from one PMP (*N* = 7; 4 females; 3 males), in which participant ages ranged between 33 and 77 years. All participants were Caucasian and living with chronic, non-malignant, musculoskeletal pain in the lower back (five participants), legs (one participant) or neck (one participant).

### Pain Management Programme Overview

The PMP was a 6 week programme, comprising two 4-h sessions per week, for adults with non-malignant chronic pain. The PMP was delivered face-to-face in a Secondary Care setting (Milton Keynes University Hospital) in the United Kingdom by a multidisciplinary team of pain management specialists. The PMP was undertaken in accordance with the Recommended Guidelines for Pain Management Programmes for Adults (26). The content of the PMP included pain psychoeducation and teaching on biological mechanisms of pain, goal setting, activity pacing, stress management, relaxation techniques, re-engaging with physical activity, cognitive behavioral approaches, self-monitoring, sleep hygiene, the benefits of social support, managing pain medication and dealing with pain flare-ups (26).

### Procedure

Participants were given a short presentation at the start of the first pain management programme session, highlighting the study's aim and design and were encouraged to ask questions about the research. Participants who volunteered to take part were provided with a consent form and written, and informed consent was obtained. Participants were informed about their right to withdraw at any point, without facing negative consequences or affecting their involvement in the PMP. It was explained that as the study was investigating naturalistic conversation in pain settings, the recording equipment (video and audio) would be set up only in the coffee room and recordings would take place during scheduled breaks between PMP teaching sessions. This was to ensure that specific personal/clinical information or Healthcare Professional-led teaching was not recorded, and patient-to-patient interactions were prioritized, in accordance with the aims of the study. Recordings could only be made when fully consenting participants were together and conversing freely without third party involvement during their PMP breaks. In total, data were viable to be collected from four 20 min coffee break sessions across 8 weeks, which resulted in 78 min of conversation of recorded data. All data gathered were stored on a password-protected laptop and external hard drive. At the end of the study, the participants were fully debriefed and signposted to organizations offering further support with managing chronic pain.

### Data Preparation and Analysis

Recordings were transcribed using Jeffersonian transcription techniques (27) and were analyzed using CA. Jeffersonian transcription method differs from verbatim transcription as it highlights talk in greater detail to understand vocal nuances, which are then included in the analysis (See Table 1 for transcription symbols). Jefferson (27) organizes in-talk interaction transcription convention into five categories: (1) transcript layout, (2) temporal and sequential relationships, (3) speech delivery, which includes tone, pitch, tempo, and emphasis, (4) metacommentary and uncertain hearings and (5) representation of non-verbal speech such as laughing and crying. Data was transcribed in full for all conversational interactions.

**Table 1:** Transcription symbols adapted from Jefferson (27).

**Timing of utterances**		
Equals sign	=	No interval between adjacent utterances
Time in single brackets	(0.5)	Length in tenths of seconds of pause in the talk
Full stop in single brackets	(.)	Pause that is less than one tenth of a second
Left hand square bracket	[	Beginning point of an overlap in utterance
Right hand square bracket	]	Ending of an overlap in utterance
**Speech delivery symbols**		
Colon	:	Extension of a sound or syllable it follows
Full stop	.	Stopping fall in tone. Does not necessarily indicate and end of a sentence
Comma	,	A continuing intonation
Question mark	?	Rising inflection, not necessarily a question
Exclamation mark	!	Animated tone, not necessarily an exclamation
Single dash	-	Halting or abrupt cut off to a word
Upward arrow	↑	Rise in pitch
Downward arrow	↓	Fall in pitch
Underline	Emphasis	Talk that is emphasized
Capital letters	LOUD	Talk that is louder than the surrounding
Degree sign	°quiet°	Talk that is quieter than the surrounding
Heh	Heh	Aspiration or laughter
‘h' in brackets	(h)	Noticeable aspiration or laughter within speech
Full stop before ‘h'	.h	Inbreath
h or series of hhh	h/hhh	Outbreath
Lesser than/greater than signs	> talk <	Talk delivered at greater speed
Greater than/lesser than signs	< talk >	Talk delivered at slower speed / elongated talk
Empty bracket	()	Inaudible transcript
Word or phrase in brackets	(talk)	Transcription in doubt
Words in double brackets	((laughs))	Description of sound

All identifiable features such as names and places were anonymised during the transcription process. Transcription was carried out independently by the first and second author. Final transcription was agreed upon by both authors through discussion and clarification of discrepancies by repeated hearings of audio data, supplemented by video data if required. Once transcription was finalized, all authors independently carried out an analysis to identify speech patterns within the data. Comparison of analysis patterns were discussed within the research group and commonalities and differences identified. The final analysis framework was therefore the result of both independent analyses and group discussion and was confirmed only when agreement was reached by all authors (28). The representativeness of the framework was further evaluated by presenting the final data to a CA specialist with significant experience of discursive psychology approaches. Through discussion, all discrepancies were resolved until consensus was reached amongst authors and the final coding framework of speech patterns was finalized.

### Conversation Analysis: Theoretical Stance and Key Conversational Mechanisms

CA aims to examine the sequence organization of talk and the social actions that it can achieve (29). It is grounded in the ethnomethodology theoretical framework and explores the detailed organization of interactions in a naturalistic setting (25). The most basic mechanism explored in CA revolves around turn-taking (30). Like other forms of coordinated activities, conversations require turn-taking to ensure organization between participants (30). Therefore, the validity of CA results emerges from focusing on how each successive turn provides evidence for how the next speaker interprets and understands the speaker (31). Additionally, Bateson (32) argues that it is possible to frame a conversation as serious or playful by signaling to the other members the playful nature of the speech. When conversations are framed within a play frame (32) participants are aware that the ongoing conversation is defined as play and participants in the conversation should interpret it like this. Finally, backchannel responses and the display of positive politeness are common in everyday conversations. Positive politeness (33) is expressed by showing similarities amongst each other and expressing appreciation and understanding of others' experiences (34). Additionally, agreement and solidarity toward a speaker is also observed through repetition of speaker's thoughts and feelings (33). Backchanneled minimal responses such as “mhmm” and “yeah” can demonstrate a form of agreement between participants (35). These mechanisms observed in conversation all help understand and frame the sequence and dynamics between individuals while interacting with each other.

## Results

**Figure 1:**
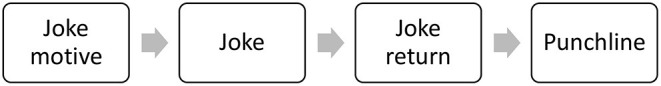
Conversational humor speech pattern.

**Figure 2:**
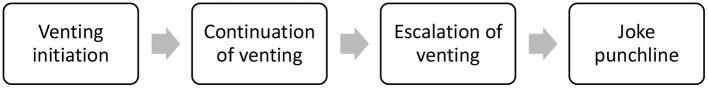
Venting cycle speech pattern.

The results of this study demonstrated that two key conversational mechanisms were active when PLwCP were interacting with each other and exploring their mutual experiences of chronic pain (see Figures 1, 2):

1. *Conversational humor*: The use of humor in naturalistic patient-to-patient conversation to share and validate pain experiences.
2. *The Venting cycle*: Catharsis of negative emotions and difficult experiences through expressing personal concerns which are received, expanded, and concluded by listeners in patient-to-patient interactions.

### Conversational Humor

Conversational humor was employed by all participants when directly discussing their personal pain experiences and pain management. The humor followed a turn-taking sequence: participants set up a joke by providing a contextual “motive,” which led into the initiation of a joke, triggering an interactive response (joke return) and the humor sequence was then closed by a punchline. Exemplars of conversational humor are demonstrated in the three extracts presented below (see **Tables 3**–**6**).

#### Extract 1: Conversational Humor

In Extract 1 (Table 2) a conversation is undertaken between three participants who joke about the amount of medication they need to take as individuals who experience chronic pain.

**Table 2:** Extract 1: Joking cycle.

**Line number**	**Pseudonym**	**Conversation text**	**Conversation analysis**
1	Tyrone:	Girlfriend's got ai ai uh ti tendonitis and arthritis(.)	Set up of joke (Motive)
2		and I think she's taking seven tablets a day and then when I	
3		turned around to ‘em and said o:h I GOT MORE TABLETS OFF UH	
4		OF A DOCTOR and she said (.) hehe	
5		>how many you taking now and I said <	Joke 1 presented
6		i'm taking 11 a da:y? now	
7	Steve:	Hehhh	
8	Tyrone:	>11 a day I'm taking < ↓ right	
9	Brenda:	16!	Conversation continues and build up to return
10	Tyrone:	16! are you	
11	Brenda:	Aye (.)	
12	Tyrone:	[Eh come ‘ere come ‘ere]	Joke return
13	Brenda:	[I tell ‘em when I when I come in and get my tablets] I say	Joke 2 presented
14		Can I come for my sweeties? now	
15	Tyrone:	[Come ‘ere come ‘ere] eh come ‘ere	Joke 2 return
16	Steve:	[Hehhhh]	Minimal response
17	Brenda:	And I said to her >if you open me up you'll find < th	Joke punchline
18		chemist inside	
19	Steve:	I could pick you up and rattle yeh Heh	

In this extract, Tyrone sets up the conversational joke by comparing the number of tablets his girlfriend takes to himself (Lines 1–3). The joke is presented when the patient says: “I'm taking 11 a da:y? now” (Line 6). The positioning of the utterance “hehe” in both lines 4 and 7 indicates the levity recognized within the conversation (Schenein, 1972), suggesting that the conversation is set within a play frame. Brenda then initiates the joke's return by suggesting they take even more medication than Steve (Line 9). Tyrone responds with “Eh come ‘ere come ‘ere” as the joke return. Comedic miming accompanied this conversational joke return, emphasizing the nature of the joke. Brenda then begins a new joke within the same context by referring to tablets as “sweeties” (Line 14). Tyrone returns the joke and links the two jokes by again saying, “Come ‘ere come ‘ere eh come ‘ere.” Finally, both Tyrone and Brenda, who have been the core participants in the joke and joke-return cycle in this extract, contribute together to the joke's punchline. To conclude the joking, Brenda describes how they have consumed enough medication to have a chemist inside them (Line 17 and 18), and Tyrone adds to this punchline by saying: “I could pick you up and rattle yeh” (Line 19).

From this extract, it is seen that rather than making a joke directed toward each other or teasing one another, the participants joke together about their own experiences and collectively share their experiences through humor. This cycle continues until the joke punchline. In this example, both individuals contribute to the punchline. The third participant in the conversation acted as a bystander who validated the play frame and collaboratively participated in the humorous nature of the conversation by uttering verbal indicators of amusement: “hehe.” Even though Steve does not interact with the conversation in-depth, the minimal response of “hehe” acts as a positive indicator that he is paying positive attention to the speaker (36) and engaging in active listening and emotional expressions of collaborative humor.

#### Extract 2: Conversational Humor

The extract in Table 3 demonstrates a second exemplar which uses the same conversational humor speech pattern. In this extract, patients joke about trialing medications and medical marijuana. Four participants are present in this conversation. However, only two (Gemma and Steve) are engaging with each other, and the other two are bystanders who are listening to the conversation.

**Table 3:** Extract 2 Joking cycle.

**Line number**	**Pseudonym**	**Conversation text**	**Conversation analysis**
211	Gemma:	That's through (.) London it's through uh King's College in	Set up of joke (Motive)
212		London that's through °so yeah I'm on trial for that°	
213	Steve:	°Like you're being treated like a guinea pig for that so°	
214	Gemma:	Yeah h. but that was the step I had to take	Continuation of joke set up
215		[No other medicine was working]	
216	Steve:	[Yeah I'm just saying you had no other choice]	
217	Gemma:	And I was like well if it's going to make me a < little bit> better	
218		then I'll give it a go	
219		So my postman arrives with my black bag	Joke 1 presented
220	Brenda:	Hehh	Minimal responses
221	Steve:	Hehhh	
222	Gemma:	It's my medication so I said as long as I-	Setup of return
223	Steve:	You mean a suitcase?	Joke return
224	Gemma:	Yeah ↑you meet a bloke around the back of an alley	Joke 2 presented
225	Steve:	Hehh	Minimal responses
226	Brenda:	Hehh	
227	Tyrone:	Yeah	
228	Gemma:	So yeah Yeah hehhhh I'd get stopped by the police.	Joke return
229	Steve:	Hehh	Minimal response
230	Gemma:	!They're prescription it's alright hehh	Joke punchline
231	Steve:	You know I'm smoking marijuana but eh hhh given to me by my GP hehh	

Gemma provides the joke's motive by discussing a medical trial program that she is on through a University. Steve demonstrates empathetic engagement toward the situation in lines 213 and 216 as Gemma describes that she is on this program as no other treatment is working. Gemma then initiates the joke in describing a postman bringing the medication: “so my postman arrives with my black bag” (Line 219). The minimal response by Brenda and Steve with the exhaled “hehh” utterance signifies the play frame and joke acknowledgment. In saying “You mean a suitcase?” (Line 223), Steve provides a return to Brenda's joke. This joke adds to the empathetic response that was given to Gemma by Steve earlier in the conversation, demonstrating social support through the use of conversational humor. The joking cycle continues with Gemma initiating another joke as a response to the previous joke return: “Yeah ↑you meet a bloke around the back of an alley” (Line 223). Once again, the other two participants' minimal responses indicate conversational engagement, and they participate in validating the collaborative humor within the conversation (Lines 225 and 226). Gemma continues the joke: “So yeah Yeah hehhhh I'd get stopped by the police.” (Line 227) and finally, both Gemma and Steve contribute to the punchline. Gemma says, “they're prescription it's alright hehh” referring to the police catching the medication, and Steve extends this punchline by adding “You know I'm smoking marijuana but eh given to me by my GP.” As the joke was steering toward illegal medication, Steve adds to the punchline by adding ironic clarification: in the hypothesized situation built within the joke he suggests that they have marijuana but it has been medically prescribed.

Similar to Extract 1, this extract demonstrates a conversational humor speech pattern that goes back and forth between participants and ends with a punchline. In this extract, the participant who provided the joke's motive was also the one to provide a punchline. Participants who were listening to this conversation provided minimal responses to demonstrate positive attention to the speakers, which demonstrates a sense of support and empathy, highlighting the collaborative nature of the humor.

#### Extract 3: Conversational Humor

The third extract presented in Table 4 follows the same conversational sequence that has been observed in the previous two extracts: Motive-Joke-Joke return-Punchline. However, the difference in this extract is that rather than the collective joking that was observed in Tables 2, 3, the joke in this extract is directed at a specific participant (Victor). Where Extracts 1 and 2 presented collaborative humor and collective joking about the experiences of living with pain was demonstrated, in Extract 3 the joke is directed at another participant (Victor), initiating an intervention from a healthcare professional. This intervention functioned to ensure that no participants felt victimized by other participants and rather that the play frame of the conversation was mutually agreed upon.

**Table 4:** Extract 3 Joking cycle.

**Line number**	**Pseudonym**	**Conversation text**	**Conversation analysis**
4	Victor:	Yeah uh my limb is swollen at the ↓moment	Set up of joke (Motive)
5	Brenda:	Really?	
6	Victor:	Yeah:	
7	Brenda:	Have you got anything for it?	
8	Victor:	Just pain killer is enough heh hhh	
9	Tyrone:	Yeah he's got an extra leg to change over	Joke 1 presented
10	Helen:	Hehhh	Minimal responses
11	Tyrone:	Hehh	
12	Brenda:	Send one over here then Victor when you find one, will you?	Joke return
13	Tyrone:	He's got two spare ones so when he get when he gets pain in	Joke 2 presented
14		one=	
15	Brenda:	=Yeah yeah take it off=	Joke punchline
16	Tyrone:	=Yeah	
17	HCP:	.h Look at this sense of humor who would have thought in	Intervention
18		Week 1 you would have been laughing about having (.)	
19		about having chronic pain. And here we are sat here:	
20	Brenda:	I know=	
21	HCP:	=I know it's great knowing it's meant as a joke there's no	
22		malice in it	
23	Tyrone:	No no no no no	Acknowledgment of play frame

*HCP, Healthcare Professional*.

From the extract, it is suggested that there is no malice intended. On the contrary, the teasing might be a form of building relations and using jokes as a coping mechanism to deal with chronic pain. Victor provides the motive for the joke by discussing his swollen limb (Line 4). After Victor says, “Just pain killer is enough hhh” (Line 8) suggesting that his situation is not too serious, and a pain killer is enough to treat it, Tyrone initiates the joke: “Yeah he's got an extra leg to change over” (Line 9). The minimal response laughter by Helen and Tyrone in Lines 10 and 11 suggests that this conversation is within a play frame, and what has been said should be interpreted as a joke. Brenda returns the joke in line 12, and Tyrone continues this return in line 13. The punchline in this conversational joke is provided by Brenda in line 15: “=Yeah yeah take it off=.” Although the participants' jokes seem targeted toward Victor, it is noted that the joke initiation is made after Victor himself downplays the severity of his situation, followed with laughter and an exhale. The uptake of this motive and its conversion to a joke then demonstrates that the participants acknowledge that Victor is understating his situation and therefore they are using conversational humor to provide support and lighten the conversation. In this extract, the healthcare professional intervenes to ensure no malice is intended toward Victor (Lines 17–19 and 21, 22). Tyrone responds with “No no no no no” further confirming that the joke was not intended to harm Victor. An intervention was required here as the joke (directed at a specific individual) could have been interpreted by Victor as malicious. Clarifying that the conversation was within a play frame allowed Victor to conceptualize the joke as it was intended, rather than experiencing it as direct teasing. Therefore, although the joke is directed at an individual, the collaborative joke was used to find positive aspects of their chronic pain experiences and lighten the collective mood.

Extracts 1–3 therefore demonstrate the stability of the first speech pattern: Motive-Joke-Joke return-Punchline, used for collaboratively for conversational humor. Using conversational humor was a sign of social support and a coping mechanism to help participants understand and make sense of their chronic pain experiences and maintain interactional levity, despite the seriousness of the pain conditions they all lived with.

### The Venting Cycle

The second speech pattern observed in the data was a venting cycle in which patients listen to each other vent about their experiences of chronic pain and pain management. The sequence of conversational venting was begun by an individual initiating an extended outline of a personal frustration, venting initiation. This was then extended or escalated further and then a joke punchline was offered either by the person venting or by a colleague to complete the venting and facilitate a change of conversational topic. The joke punchline therefore served as a pivot in conversation whereby the person who held the floor would pass their turn over to someone else (37). After the turn was passed over, the expression of negative feelings passed from one participant to another or entirely stopped. The punchline also demonstrated conversational engagement among participants, which served as a form of social support to help participants make sense of their experiences and validate their vent.

#### Extract 4: Venting Cycle

Extract 4 (shown in Table 5) presents a scenario where two patients converse about chemists and the difficulties of obtaining (correct) prescriptions. In this extract, both participants talk and listen to each other vent about their shared negative experiences of seeking pain medication:

**Table 5:** Extract 4: Venting cycle.

**Line number**	**Pseudonym**	**Conversation text**	**Conversation analysis**
173	Brenda:	Huh that's good because our chemist is absolutely (.)	Vent initiation
174		Terrible. (.) never get our medication on time: I had to ring	
175		up and complain about it=	
176	Steve:	=Yeah I was suffering from that °I was getting a lot of th	Venting continues
177		cos I was like the ones that was um: I actually ran out of	
178		patches and you know I can't do without them because-	
179	Brenda:	>Of course not <	
180	Steve:	Because you: need them for the one for the pain yes, but	
181		also there's that you get withdrawal symptoms if you're not	
182		taking them	
183	Brenda:	[In fact um-]	
184	Steve:	[They can't win though-]	
185	Brenda:	One time, I always used to say to the pharmacist the last (.)	Venting escalates
186		and when I'm down to two patches (.) >can you order them	
187		for me please <	
188	Steve:	Yeah	
189	Brenda:	I say because I don't wanna run out I'd rather-	
190	Steve:	Oh crikey NO	
191	Brenda:	I'd rather be 2 weeks I have 2 weeks supply >and then	Venting continues
192		all of a sudden < forget about it and run out but but um	
193		but um: she wasn't doing it? see	
194	Steve:	°No°	
195	Brenda:	So: my husband then complained to them about it and then	
196		the woman said oh I'll look into this and she did	
197	Steve:	Yeah	
198	Brenda:	And now the pharmacist is spot on	
199	Steve:	Sometimes >take a kick up the bum and then < heh	Joke punchline
200	Brenda:	Just like she needs u:m fireworks	
201	Steve:	Hehhhhh	Minimal response
202	Brenda	°So yeah°	
203	Steve:	Anyway	Change in turn

Brenda initiates her venting in line 173 by describing her negative experience with a chemist. Steve interrupts and shares his common experience in Line 176, which demonstrates the continuation of the vent. Steve and Brenda continue to share their negative experiences between lines 179–184. In line 183, Brenda interrupts Steve with overlapping speech and resumes her vent, showing an upgraded agreement to his venting by sharing a related experience related to his situation (lines 185–187). From lines 188 to line 197, Steve provides minimal responses such as “Yeah” (Line 188), “Oh crikey no” (Line 190) and “No” (Line 194) in response to the escalating venting by Brenda (Lines 188, 190, 194, and 197). These minimal responses allow for the venting to continue as it provided positive reinforcement and attentional acknowledgment. Steve initiates the joke punchline in line 199: “Sometimes >take a kick up the bum and then < heh” and Brenda adds to the joke: “Just like she needs u:m fireworks.” Following the joke punchline, there is a conversational pivot from a mutual vent to a more general conversation. Additionally, a change in turn taking is observed from Brenda who was the primary initiator of the venting, allowing Steve to take the floor. This extract demonstrates a Venting initiation-Continuation and escalation of venting-Joke punchline to finish the venting speech pattern. The mutual use of minimal responses allowed the individuals to continue to express their negative experiences to each other.

#### Extract 5: Venting Cycle

Demonstrating a similar speech pattern as Extract 4, the fifth extract presented in Table 6 shows an interaction between two individuals who discuss pain medication and the worsening of their pain symptoms. In this extract, one participant releases their negative emotions while the other participant listens:

**Table 6:** Extract 5: Venting cycle.

**Line number**	**Pseudonym**	**Conversation text**	**Conversation analysis**
22	Steve:	When my wife comes back with her big bag of meds and I go.	Vent initiation
23	Brenda:	Yeah	
24	Steve:	Is that all mine ?and she goes yeah. oh bugger (.) I'm just going	
25		through a um change in my medication now so I'm	Venting continues
26		going through (.) quite a bit of problems with pain and stuff so=	
27	Brenda:	=Uh huh uh huh=	Minimal response
28	Steve:	=My pain level is getting worse ↑ I started	Venting escalates
29		>it a bit at the wrong time because <	
30		this literally this week is my first week on this new thing	
31	Brenda:	Uh huh=	
32	Steve:	=And I've got to reduce and reduce and reduce and then	
33		increase the other bits h	
34	Brenda:	Ah:=	
35	Steve:	=So I'm going to be going through the alphabet anyway I'll just	Joke punchline
36		get through it ↑°hopefully°	
37	Brenda:	Ok um: the doctor gave me medication prescribed ‘em to me	Change in turn
38		this morning	
39	Steve:	Yeah	
40	Brenda:	He prescribed me [medication name] yeah	
41	Steve:	Well I've got them yeah:	
42	Brenda:	What did you say? what milligram you're on	
43	Steve:	I used to have two:	

Steve initiates the vent by discussing the amount of medication he has to take: “When my wife comes back with her big bag of meds, and I go” (Line 22) “Is that all mine? and she goes yeah. oh bugger (.)” (Line 24). He discusses his “pain level getting worse” and his pain escalation as a result of starting the medication at the wrong time, which demonstrates an escalation in his venting. Finally, Steve provides the joke punchline: “=So I'm going to be going through the alphabet anyways I'll just Get through it ↑°hopefully°.” In this extract, Steve is the participant who is venting and he ends his own venting with a joke. Similar to the first extract, a conversational pivot occurs after the punchline as there is a change in turn taking and Brenda begins to share her experience regarding her prescribed medication.

In this extract, Brenda provides backchannel responses throughout the conversation. These include “Yeah” (Line 23), “=Uh huh uh huh=” (Line 27) and “Ah:=” (Line 34). This demonstrates that she is engaged and actively listening to Steve. Additionally, in line 42, Brenda asks, “What did you say what milligram you're on,” which indicates engagement, positive politeness and curiosity as she has followed up with a question that was related to Steve's expression of emotion. The Venting initiation-Continuation and escalation of venting-Joke punchline to finish the venting is consistent in this extract with engagement and active listening by all participants.

#### Extract 6: Venting Cycle

The final extract, shown in Table 7, presents a situation where five patients have a conversation about pain medications and medical trials. Even though five members are present in the conversation, one participant vents about their experience with medical medicine trials whilst the others actively listen.

**Table 7:** Extract 6: Venting cycle.

**Line number**	**Pseudonym**	**Conversation text**	**Conversation analysis**
133	Brenda:	I've told the doctor I don't >want to be treated as guinea pig <	Vent initiation
134	Steve:	No h	
135		right try me on this one: try me on that one: no (.) I said no	Venting continues
136		want the proper medication	
137	Gemma:	°Yeah my surgery is the same it's ridiculous°	
138	Brenda:	And >once the other day going to the doctor < and he said t	Venting escalates
139		me oh. you've got to come of this tablet you can't have it	
140		anymore and uh and my husband said (.) hang on you've	
141		just got the medication right! you can't just say to her	
142		mo she's not gonna have it=it's like he gave me morphine	
143		I've passed out with morphine patches it started off (.) With	
144		the 12 and then I've went up to 75.h and um he said to me	
145		I might have to take you off them and I said NO YOU WON'T	
146		I said I'm not going to be crawling in agony	
147	Steve:	No	
148	Brenda:	And he said to me=my doctor said to me I'm gonna try	Venting continues
149		on um morphine tablets ↓oh my God. the first week I was	
150		supposed to take a tablet in the evening the second week	
151		I'm supposed to take two tablets on the first week I was sick	
152		all: week with them	
153	Steve:	Well yeah	
154	Brenda:	And then my ankle from here down blew up like that	
155		I couldn't get off the settee I couldn't my husband	
156		couldn't even help me to get off and I saw the doctor about it	
157		he said I want you to stop them immediately and I said	
158		I did. Don't you worry	
159	Steve:	[Yeah hehh]	
160	Gemma:	[Hehh]	
161		To touch them you are lucky to be al to be alive	
162	Brenda:	I said I TOOK what YOU GAVE me	
163	Gemma:	Yeah	
164	Steve:	Mm	
165	Brenda:	And I said to him I am not a guinea pig and apparently the	Venting escalates
166		drugs he gave me were for (.) depression and epileptic fits	
167		which I don't suffer with (.) there are two: two medications	
168		into one and I don't even suffer from epileptic fits or	
169		depression	
170	Gemma:	Yeah h	
171	Steve:	.h No	
172	Kate:	So: I take it that it-	
173	Tyrone:	With the doctor I'd-	
174	Brenda:	See! so what's the point!	
175	Tyrone:	Patients are guinea pigs >you know what I ↑mean <	Venting continues
176	Brenda:	[Try thi:s try tha:t]	Joke punchline
177	Tyrone:	[Try that like] it it might take your pain away but like I say	Joke punchline
178		it might make you looney or something like that	Joke punchline
179	Steve:	°It's it's a side effect°	Change in turn
180	Tyrone:	.h It's like that when they gave me amitriptyline	

In suggesting that they do not “want to be treated as a guinea pig” (Line 133) for medical trials, Brenda initiates the venting. Immediately Steve provides a minimal response of ‘no' (Line 134). This response indicates that there is engagement and active listening as he voices his opinion to agree with what Brenda proposed. Steve to express his negative experiences in lines 135, 136, and Gemma gives an upgraded agreement indicating collaborate support for to the context and content of the venting: “Yeah my surgery is the same it's ridiculous” (Line 137). Brenda continues and escalates her venting about her medication and pain experiences from Line 138−169. Throughout this section, participants provide minimal responses and agreement to Brenda, who is holding the floor. These responses include laughter, “Yeah hehh” (Line 159) and minimal agreement such as “Well yeah” (Line 153), “Yeah” (Line 163), and “Mm” (Line 164). These responses suggest agreement, engagement and responsiveness from the participants listening to their colleague speak about their experience. Additionally, Tyrone repeats Brenda's negative sentiment about patients being guinea pigs in Line 175. This further demonstrates displays of agreement toward Brenda, which provides the incentive for Brenda to continue her vent.

Participants are demonstrating recognition of the need to move beyond the vent and therefore the joke punchline was initiated by Brenda, who began the vent: “Try thi:s try tha:t” (Line 176) and Tyrone adds to the punchline: “Try that like it it might take your pain away but like I say it might make you looney or something like that” (Line 177, 178). Again, the joke punchline pivots the conversation from a participant venting to Tyrone taking the floor and speaking about his experience in comparison to Brenda's experience. This is acknowledged with a minimal “hehh” (Line 181) by Gemma, which demonstrates the ongoing play frame of the conversation.

The venting cycle extracts have demonstrated that pain venting cycles reliably occur among patient-to-patient interactions. The speech pattern observed in venting cycles is as follows: Vent initiation-Continuation of venting-Escalation of venting-Joke punchline, but it can be both collaborative venting, or venting by a single person, supported by others. Actively listening to other participants' venting adds to the sense of shared pain experience and allows for an individual's feelings to be validated by individuals with similar experiences.

## Discussion

Results from this observational study demonstrated that patient-to-patient conversations within pain management settings were employing two specific interactional mechanisms, identified via conversation analysis. These approaches to communication were: (1) conversational humor and (2) a venting cycle. These patterns in speech demonstrated how CP patients expressed their pain experiences and supported each other through constructive interpersonal interactions. Both conversational mechanisms involved the use of humor, collaborating together to describe pain experiences with levity. These conversations allowed PLwCP space to express their negative emotions, before ending this cathartic vent with a light-hearted punchline, which served to pivot the conversation toward other contributors and new topics. Although the humor was targeting difficult experiences of pain and pain management, the positive reception and adoption of humor within the group indicated that the humor facilitated the discussion of sensitive topics in a socially supportive environment.

The results of this study showed how fundamental the use of humor is in interpersonal interactions, even within a pain management setting. The deliberate application of humor to difficult situations has been demonstrated in other chronic illness populations; after radiotherapy, humor enabled people with cancer to cope better with their health (38). Similarly, quantitative assessment of humor use within pain, has suggested that PLwCP may employ humor to gain perspective on their pain and distance themselves from their situation by accepting the negative emotions that common when living with chronic pain (39). In confirmation, a recent narrative review on pain and humor suggested that humor serves to improve pain coping and minimize emotional distress (40). Humor in pain management settings may therefore act as a method of bolstering pain-related resilience and reducing pain catastrophising (41). Certainly, in this study, the deliberate use of humor was evidenced through consistent use of jokes and delivery of punchlines, even when these may be least expected, for example, after venting and describing difficult pain-related situations. Finding the humor in chronic pain is indicative of highly subjective, black humor, which is mutually understood and facilitated by participants' common experiences, but which may not fully translate to others without pain or an understanding of chronic pain. So-called “black humor” or “gallows humor” is recognized as having therapeutic value, particularly when used by individuals facing trauma or challenging circumstances in health settings (42). Indeed, in clinical contexts, such self-deprecating humor has been found to boost psychological well-being, despite its dark(er) content (43), suggesting it is adaptive in the pain management context.

The theoretical underpinning of the humor evidenced in the current study provides an interesting disconnect between our findings and previous interpretations of humor in pain management. Previous research, using a cross-sectional quantitative assessment of humor, pain and psychological well-being has been interpreted as indicating that humor is a distancing mechanism, a way of making space between chronic pain and oneself as a method of self-protection (39). However, the real-life conversational mechanisms demonstrated in this study did not suggest that interactive humor was a distancing mechanism. Indeed, an approach-based pattern was found; in the naturalistic interactions we recorded, there was a clear willingness to “vent” by giving detailed descriptions about one's pain experience, setting up a conversational context in which PMP group members actively chose to *enter into* the discussion and to collaborate in joking about pain. This suggests a willingness to approach rather than avoid the pain situation that is described by their colleague and a willingness to approach one's own pain when venting.

The difference between our findings here and that of Ramírez-Maestre et al. (39) may be explained by the “optimal matching hypothesis” (9). In the current study, all participants already had a mutual understanding of chronic pain and therefore the shared pain experience was a commonality between the group, allowing for psychological safety in approaching this difficult topic (44) removing the need to employ distancing. Therefore, patient-to-patient interactions in PMPs were evidencing an *affiliative humor style*, using humor to enhance one's relationship with others (45). This is powerful evidence that interpersonal, conversational humor in PLwCP is not aggressive, self-enhancing, self-defeating or distancing. It is constructive, collaborative, communicative and creates warm appreciation of others within the group, respecting the shared mutual understanding of chronic pain. Further research is needed to establish whether this humor style only worked because of group dynamics or whether it can be identified more widely. Where “dark humor” is employed, there is the risk that such humor could be misconstrued, causing unease, alienation or even perceived insult (46). The participants in this study were all PLwCP, which provided a certain level of implicit understanding of pain experiences that is likely not there in interactions between different patient populations or between people with and without CP.

The prosocial nature of the humor demonstrated in this study was unusual in that it employed mutual sarcasm, with the sarcasm applied not to a person, but to the context under discussion-chronic pain itself. Wider literature suggests that the type of humor is important when addressing health conditions (47, 48) for example, mocking and sarcasm are typically viewed negatively (38). However, in this study, the participants used sarcasm that was received positively and was directed toward pain rather than a person or their behavior. The sarcasm highlighted the ridiculousness of the situation (for example, being prescribed numerous medications) rather than ridiculing the speaker, suggesting that the target of the humor may be more important compared to humor type in interactions between PLwCP. The current study demonstrated one incidence (Extract 3) when use of sarcasm came closer toward being associated with an individual, rather than pain more generally. In that instance, the healthcare professional stepped in to maintain the prosocial balance in the humor, though the participants in the conversation showed strong awareness of the need to keep their conversational humor inclusive. It is possible therefore that HCPs may benefit from additional awareness and training in the value of humor within the pain management setting, to respond to and encourage such humor effectively.

The venting cycle that was demonstrated in this research found that PLwCP were active listeners to their colleagues' distress, using positive minimal response (mmm, yeah) and interjecting shared experiences to demonstrate empathetic listening. This shows that group interactions in the PMP setting are building pain coping, as has also been shown in chronic pain support groups (41). Yet, the venting and the humor which concludes the vent cycle are not a distraction away from pain, posited as a primary theoretical explanation for the benefits of humor in pain management (40), nor are they methods of promoting cognitive reappraisal of pain (49). Instead, the venting cycle, is further evidence of the power of social support to improve pain coping and pain management (50). The vent is allowed to continue by the listeners, who help the speaker through the expression of their difficult experience. Therefore, the vent is acting as a method of emotion regulation, which can help to mediate the relationship between negative emotions and anxiety or depressive disorders (51).

### Limitations and Future Recommendations

The recruitment for the current study was only from one of the three potential PMPs, and participants were recruited from one geographical site. Whilst this may limit generalizability of the findings, this is the first study, to the authors' knowledge, that uses CA to examine conversations between CP patients rather than between a health professional and CP patients. Significant efforts were made to ensure that naturalistic conversation could be recorded, but the presence of audio-visual recording equipment inevitably limits this. Future research could aim to hold a focus group with participants after the conversational mechanisms are identified via CA, to allow participants to validate the observations. Future research could aim to confirm with participants *post-hoc* the reasoning for the conversational humor and venting cycle. The findings need to be replicated in comparable settings and confirmed *post-hoc* with CP patients before the universality of such mechanisms can be reliably confirmed. Additionally, the cultural context of the current study was within a British, Caucasian sample, and it is likely that individuals from other cultures may not employ or perceive humor as a positive conversation mechanism which nurtures social support. It is possible that without a common understanding of chronic pain, pain humor could be misperceived as exclusionary or an insult (46), which would jeopardize group cohesion and clinical outcomes. Future research could assess patient-to-patient interactions with a more culturally diverse sample, and across a variety of inpatient and outpatient pain management settings to identify commonalities and variation in pain humor usage.

## Conclusions

Humorous interpersonal interactions about chronic pain act as an important forum for social support-building between members of a PMP. Such dynamic interactions referenced the mutual experience of chronic pain and fostered common humor, helping people living with chronic pain to make sense of their pain in a prosocial atmosphere. When negative emotions were strongly expressed through venting, responses were empathetic with active listening used to validate the negative emotions, allowing for a cathartic expression of personal challenges. Patient-to-patient interactions within the PMP were therefore strongly prosocial and mutually inclusive, evidencing that the socially communicative context fostered during PMPs has significant power to facilitate enhanced clinical outcomes and group cohesion.

## Data Availability Statement

The raw data supporting the conclusions of this article will be made available by the authors, without undue reservation.

## Ethics Statement

The studies involving human participants were reviewed and approved by NHS Ethics REC: 13/WM/0214. The patients/participants provided their written informed consent to participate in this study.

## Author Contributions

KF and SP: conceptualization of research. KF: data collection. KF, KA, and AM: data analysis and drafting the manuscript. KF, KA, AM, and SP: editing and review of manuscript. All authors contributed to the article and approved the submitted version.

## Conflict of Interest

The authors declare that the research was conducted in the absence of any commercial or financial relationships that could be construed as a potential conflict of interest.

## Publisher's Note

All claims expressed in this article are solely those of the authors and do not necessarily represent those of their affiliated organizations, or those of the publisher, the editors and the reviewers. Any product that may be evaluated in this article, or claim that may be made by its manufacturer, is not guaranteed or endorsed by the publisher.
